# Effects of α-Lipoic Acid on Phagocytosis of Oligomeric Beta-Amyloid_1–42_ in BV-2 Mouse Microglial Cells

**DOI:** 10.3389/fnagi.2021.788723

**Published:** 2022-01-12

**Authors:** Chih-Yuan Ko, Jian-Hua Xu, Yu-Wei Chang, Yangming Martin Lo, James Swi-Bea Wu, Wen-Chung Huang, Szu-Chuan Shen

**Affiliations:** ^1^Department of Clinical Nutrition, The Second Affiliated Hospital of Fujian Medical University, Quanzhou, China; ^2^Department of Respiratory and Critical Care Medicine, The Second Affiliated Hospital of Fujian Medical University, Quanzhou, China; ^3^School of Public Health, Fujian Medical University, Fuzhou, China; ^4^Respiratory Medicine Center of Fujian Province, Quanzhou, China; ^5^Department of Tumor Surgery, The Second Affiliated Hospital of Fujian Medical University, Quanzhou, China; ^6^Graduate Program of Nutrition Science, National Taiwan Normal University, Taipei, Taiwan; ^7^Institute for Advanced Study, Shenzhen University, Shenzhen, China; ^8^Graduate Institute of Food Science and Technology, National Taiwan University, Taipei, Taiwan; ^9^Graduate Institute of Health Industry Technology, Chang Gung University of Science and Technology, Taoyuan, Taiwan

**Keywords:** α-lipoic acid, microglia, oligomeric beta-amyloid, phagocytosis, Alzheimer’s disease (AD)

## Abstract

**Background and objectives:** This study aimed to investigate the enhancing effect of vitamin-like alpha-lipoic acid (ALA) on phagocytosis of oligomeric beta-amyloid (oAβ)_1–42_ in BV-2 mouse microglial cells.

**Methods:** An *in vitro* model was established to investigate phagocytosis of oAβ_1–42_ in BV-2 cells. Transmission electron microscopy images indicated that the morphology of prepared oAβ_1–42_ was spherical particles. BV-2 cells treated with ALA were incubated with 5(6)-carboxyfluorescein-labeled oAβ_1–42_ (FAM-oAβ_1–42_) for 24 h, followed by flow cytometer analysis, western blotting, real-time quantitative PCR, and immunocytochemistry (ICC) analysis to assess the *in vitro* phagocytosis ability of oAβ_1–42_.

**Results:** Alpha-lipoic acid significantly increased messenger RNA (mRNA) expression of the CD36 receptor in BV-2 cells. ICC analysis showed that ALA significantly elevated CD36 protein expression in BV-2 cells both with and without oAβ_1–42_ treatment. Results from the flow cytometry analysis indicated that the CD36 receptor inhibitor significantly attenuated ALA-promoted phagocytosis of FAM-oAβ_1–42_ in BV-2 cells. Moreover, ICC analysis revealed that ALA caused the translocation of peroxisome proliferator-activated receptor-γ (PPAR-γ), which is known to regulate the expression of CD36 mRNA in BV-2 cells. ALA also elevated both the mRNA and protein expression of cyclooxygenase-2 (COX-2), which is a key enzyme involved in the synthesis of 15-deoxy-^Δ12,14^-prostaglandin J2 in BV-2 cells.

**Conclusion:** We postulated that ALA enhances oAβ_1–42_ phagocytosis by upregulating the COX-2/15-deoxy-^Δ12,14^-prostaglandin J2/PPAR-γ/CD36 pathway in BV-2 cells. Finally, future studies should be conducted with an *in vivo* study to confirm the findings.

## Introduction

Alzheimer’s disease (AD) is an irreversible neurodegenerative disease with cognitive impairment that accounts for more than half of patients with dementia (Knopman et al., 2021). To date, while the complexity of neurodegeneration continues to be explored and recognized, AD has been characterized in part by excessive extracellular beta-amyloid (Aβ) plaque accumulation, the presence of intracellular tau-containing neurofibrillary tangles, and neuroinflammation in the brain (Erika et al., 2018; Rai et al., 2020; Knopman et al., 2021; Singh et al., 2021). In the brains of patients with AD, synaptic loss can precede neurodegeneration, brain tissue gradually shrinks, which, in turn, leads to a decline in memory and learning abilities of patients, eventually causing dementia (Blennow et al., 2006; Srivastava et al., 2019; Tripathi et al., 2019). Synaptic alterations are actually a critical part of neurodegeneration that are strongly correlated with the morphological lesions of AD, neurotoxicities of tau and Aβ, and cognitive impairment, which induce various neurotoxic effects such as increased inflammation, increased oxidative pressure, and subsequently damages the nerve synapses of the brain (Jack et al., 2019; Srivastava et al., 2019; Knopman et al., 2021). These factors gradually alter the microenvironment system of the brain, which becomes unconducive to the survival of nerve cells. In addition, inflammation plays a hallmark role in progression of AD. Aβ accumulation facilitates activation of immune cells including microglia, astrocytes, and macrophages. Remarkably, when microglia are activated in AD, activated microglia contribute to degraded Aβ accumulation through autophagy or clearance. Additionally, chronically activated microglia stimulating to the release of cytokines, which initiate a proinflammatory cascade and subsequently induce synaptic alterations (Pomilio et al., 2020; Knopman et al., 2021). There is a strong correlation between oligomeric Aβ (oAβ) accumulation in the brain and cognitive ability (Benilova et al., 2012). The oAβ strongly inhibits long-term potentiation of the hippocampal gyrus and causes damage to the nerve synapse, which eventually results in memory deficits in the patient (Walsh et al., 2002; Haass and Selkoe, 2007; Querfurth and LaFerla, 2010; Benilova et al., 2012). Therefore, oAβ is a crucial cause of occurrence of AD; however, the mechanism is still unclear.

Microglial autophagy may be potentially affected for treating AD (Pomilio et al., 2020). Evidence has highlighted that autophagy and antioxidants play core roles in AD (Liu et al., 2015). According to the theory of oxidative stress, numerous antioxidants are associated with the prevention of progression of AD (Su et al., 2008). For example, 15-deoxy-^Δ12,14^-prostaglandin J2 (15-deoxy-^Δ12,14^-PGJ2), which is produced by cyclooxygenase (COX), has been widely accepted as an anti-inflammatory prostaglandin and its anti-inflammatory mechanism is related to peroxisome proliferator-activated receptor-γ (PPAR-γ) (Scher and Pillinger, 2005). PPAR-γ is the transcription factor of CD36 messenger RNA (mRNA). When the activity of PPAR-γ increases, the transcription of CD36 mRNA increases by cells (Shimizu et al., 2008; Yamanaka et al., 2012). Bujold et al. (2009) successfully linked the mechanisms of COX-2/15-deoxy-^Δ12,14^-PGJ2/PPAR-γ/CD36 to elucidate in atherosclerosis. This is a critical mechanism pathway for this study.

The vitamin-like compound, alpha-lipoic acid (ALA), is a thioctic acid- and sulfur-containing organic compound that is a natural biological antioxidant (Packer et al., 1995). It can combat various chronic diseases such as diabetes, its related complications, and hypertension (Thirunavukkarasu et al., 2004; Ko et al., 2021). ALA has also been proven to affect the central nervous system by penetrating the blood–brain barrier (BBB) (Packer et al., 1995). Thus, ALA has been used to study numerous neurodegenerative diseases. One study showed that administering 600 mg/day of ALA to patients with AD can delay the progression of AD (Hager et al., 2007). Study on ALA and AD has largely focused on antioxidation, anti-inflammation, and the promotion of brain glucose metabolism. However, there are limited investigations on the use of ALA to promote the clearance of Aβ by microglia, thereby reducing the neurotoxic effects of Aβ-induced brain oxidative stress, inflammation, and glucose uptake resistance (Holmquist et al., 2007; Maczurek et al., 2008; Farr et al., 2012; Dos Santos et al., 2019; Kim et al., 2019; Choi et al., 2020). Therefore, we evaluated the alleviating effect of ALA on progression of AD and investigated the possible mechanism of ALA in promoting phagocytosis of BV-2 cell oAβ_1–42_ and its association with the COX-2/15-deoxy-^Δ12,14^-PGJ2/PPAR-γ/CD36 pathway.

## Materials and Methods

### Cell Culture

The mouse microglial BV-2 cell line used in this study was kindly donated by Professor Fu’s laboratory of the College of Medicine, National Taiwan University. BV-2 cells were cultured in an F12 medium without phenol red, containing 100 U/ml penicillin, 100 g/ml streptomycin, and 10% fetal bovine serum (FBS) at 37°C and 5% carbon dioxide (CO_2_) (Lu et al., 2007).

### Identification of oAβ_1–42_ Aggregation Pattern

The Aβ_1–42_ monomer was purchased from Biopeptide (San Diego, CA, United States). The preparation of oAβ_1–42_ was adopted by the previous study (Yang et al., 2011). The appearance of the prepared oAβ_1–42_ was observed using JEM-1200EX II transmission electron microscopy (TEM) (JEOL, Tokyo, Japan). OAβ_1–42_ was first stained with 1% phosphotungstic acid for 2 min and placed on a 200-mesh copper net for observation. Then, freshly dissolved and fibrillary Aβ_1–42_ was compared to observe whether oAβ_1–42_ had an ideal appearance. In addition, the molecular weight of oAβ_1–42_ was analyzed using XL-A analytical ultracentrifugation (AUC) (Beckman, Palo Alto, CA, United States); the sample was centrifuged at 60,000 rpm and the orientation of the Aβ_1–42_ peptide was detected at a wavelength of 280 nm (Yang et al., 2011). Results of AUC were fitted to the continuous c(s) distribution model using the SEDFIT software^1^ and the molecular weight and percentage of aggregation of oAβ_1–42_ were estimated.

### Oligomeric Beta-Amyloid_1–42_ Uptake Analysis and Quantification

The fluorescent molecule 5(6)-carboxyfluorescein (FAM) was used as a calibrator to explore the uptake of oAβ_1–42_ in BV-2 cells. When cells uptake oAβ_1–42_, calibrated by FAM, they were excited by a light with a wavelength of 488 nm and emitted green fluorescence of approximately 517 nm. This fluorescence was detected and accurately quantified using the LSRFortessa Flow Cytometer (BD Biosciences, Franklin Lakes, NJ, United States).

### Real-Time Quantitative PCR Analysis

According to the instructions of the manufacturer, the total RNA of cells was extracted using the Direct-zol RNA MiniPrep Kit (Zymo Research, Irvine, CA, United States). RNA concentrations were determined by the ND-1000 Spectrophotometer (NanoDrop^®^). Complementary DNA (cDNA) was synthesized by adding preisolated RNA and RNA to the cDNA EcoDry™ Premix (Oligo dT; Clontech Laboratories Incorporation, Mountain View, CA, United States). Real-time quantitative PCR was used to analyze genes such as scavenger receptor A1 (SR-A1), scavenger receptor B1 (SR-B1), CD36, receptor for advanced glycation endproducts (RAGE), COX-2, and glyceraldehyde 3-phosphate dehydrogenase (GAPDH). Sequences for the primer sets used in quantitative PCR for each gene are given in Table 1. The amplification reactions were performed using the StepOnePlus™ Real-Time PCR System (Applied Biosystems, Foster City, CA, United States). Data were described using the relative quantification method 2^–ΔΔCt^.

**TABLE 1:** Primer sequences for real-time PCR (RT-PCR).

Gene name	Forward (5′-3′)	Reverse (5′-3′)
SR-A1	CTGGACAAACTGGTCCACCT	TCCCCTTCTCTCCCTTTTGT
SR-B1	TTTGGAGTGGTAGTAAAAAGGGC	TGACATCAGGGACTCAGAGTAG
CD36	GAACCACTGCTTTCAAAAACTGG	TGCTGTTCTTTGCCACGTCA
RAGE	ACTACCGAGTCCGAGTCTACC	GTAGCTTCCCTCAGACACACA
COX-2	AGACAGATCATAAGCGA GGACC	CCTCTCCACCAATGACCTGATATT
GAPDH	TGCACCACCAACTGCTTAGC	GGCATGGACTGTGGTCATGAG

### Western Blot Analysis

The procedure for Western blot was carried out according to our previous study (Chen et al., 2021). Briefly, the protein concentration in the cell extract was determined using a Bio-Rad protein assay dye reagent (Richmond, Virginia, VA, United States). Aliquots of the supernatant containing 50 μg of protein were separated using sodium dodecyl sulfate polyacrylamide gel electrophoresis (SDS-PAGE) and electrophoretically transferred onto polyvinylidene difluoride membranes. The membranes were then incubated with anti-COX-2 (1:1,000; Cell Signaling Technology, Danvers, MA, United States) and anti-GAPDH (1:5,000; Signalway Antibody Corporation, College Park, MD, United States) antibodies at 4°C overnight. Next, the membranes were incubated with antirabbit immunoglobulin G and washed thrice for 5 min each time. The final images were detected and captured using the UVP Biospectrum Imaging System (Level, Cambridge, United Kingdom). Finally, all the relevant protein expressions were normalized with GAPDH.

### Immunocytochemistry

A total of 4 × 10^4^ cells were cultivated on a cover glass. After 24 h, the prepared oAβ_1–42_ was added and incubated for 24 h, fixed with 3% formaldehyde, punched with 0.1% Triton X-100, and placed in phosphate buffered saline with Tween (PBST) containing 5% bovine serum albumin (BSA) for blocking overnight. On the subsequent day, cells were stained with the primary antibody. After the invariable reaction overnight, cells were stained with a secondary antibody of fluorescent molecule Alexa 488 or 647. The nuclei were stained with 4′,6-diamidino-2-phenylindole (DAPI) and observed under an upright fluorescence microscope (Carl Zeiss, Jena, Germany, United Kingdom). Cell fluorescence intensity was analyzed using MetaMorph (Molecular Devices, Sunnyvale, CA, United States).

### Detection of Nitric Oxide

A total of 2 × 10^5^ cells were cultured in six-well plates for 24 h, replaced with 1.5 ml Dulbecco’s Modified Eagle Medium (DMEM) without FBS and lipopolysaccharides treatment, and cocultured with cells for 24 h. The cell culture fluid was collected and mixed with Griess reagent in a 1:1 manner and reacted for 15 min and absorbance was detected at 540 nm using an ELISA reader.

### Analysis of 15-Deoxy-^Δ12,14^-PGJ2

A total of 3 × 10^5^ cells were cultured in six-well plates for 24 h, replaced with FBS-free DMEM, and treated with different concentrations of ALA. The cells were pretreated with ALA for 30 min and the vehicle or prepared oAβ_1–42_ was added and cocultivated for 24 h. An ELISA reader was used to analyze the culture medium. Standard of 15-deoxy-^Δ12,14^-PGJ2 was diluted with 400 μM ALA and the 4-parameters logical curve was used to analyze the 15-deoxy-^Δ12,14^-PGJ2 concentration of the sample.

### Statistical Analyses

Values are presented as the mean ± SD using the SAS version 9.2 (SAS Institute Incorporation, Cary, NC, United States). The one-way ANOVA and Duncan’s multiple range tests were performed. *p* < 0.05 was accepted as statistically significant.

## Results

### Analysis of Transmission Electron Microscopy Appearance and Analytical Ultracentrifugation Physical Properties of Aβ_1–42_

**FIGURE 1:**
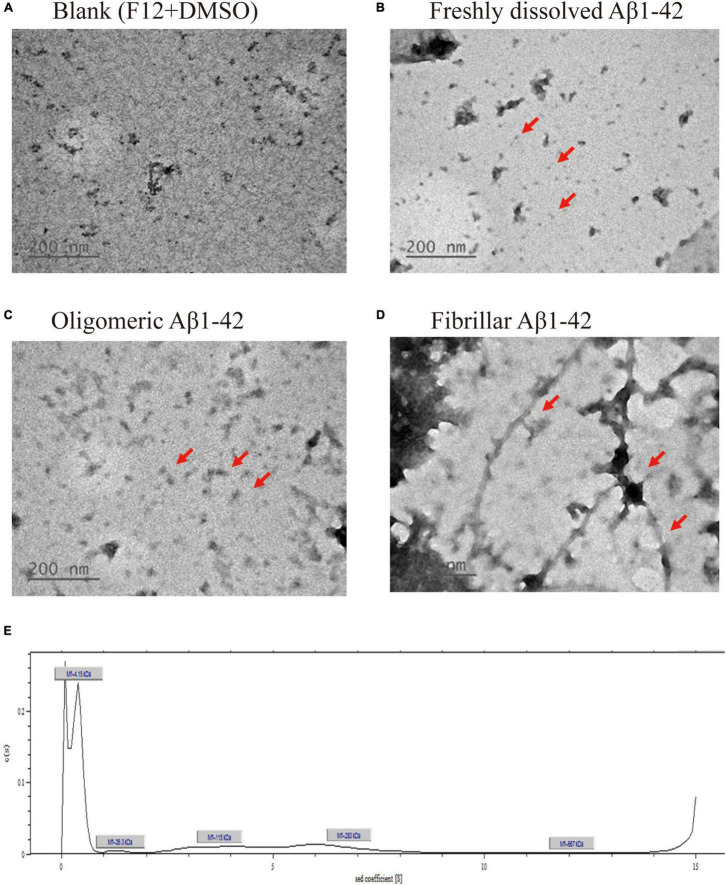
Transmission electron microscopy (TEM), morphological and analytical ultracentrifugation (AUC), and physical characterization analysis of prepared beta-amyloid (Aβ_1–42_). TEM image of **(A)** blank [F12 medium + dimethyl sulfoxide (DMSO)], **(B)** freshly dissolved Aβ_1–42_, **(C)** oligomeric Aβ_1–42_, **(D)** fibrillar Aβ_1–42_, and **(E)** Sedimentation coefficient analysis of oligomeric Aβ_1–42_ using AUC.

The appearance of oAβ_1–42_ as a blank group (Figure 1A), freshly prepared Aβ_1–42_ (Figure 1B), oAβ_1–42_ solution (Figure 1C) after 24 h of aggregation at 4°C, and fibrillary Aβ_1–42_ with an apparent fibrous production group (Figure 1D) was examined using TEM. AUC was used to analyze the sedimentation coefficient of the prepared oAβ_1–42_ and the molecular weight and friction ratio of oAβ_1–42_ were further fitted using the SEDFIT software, as shown in Figure 1E. The molecular weight of the Aβ_1–42_ monomer was approximately 4.6 kDa. Approximately, 50% of Aβ_1–42_ aggregated into an oligomeric form and three primary oligomeric forms with molecular weights of 26.3, 115, and 28 kDa were observed.

### Effect of Alpha-Lipoic Acid on Uptake of FAM-oAβ_1–42_ in BV-2 Cells

**FIGURE 2:**
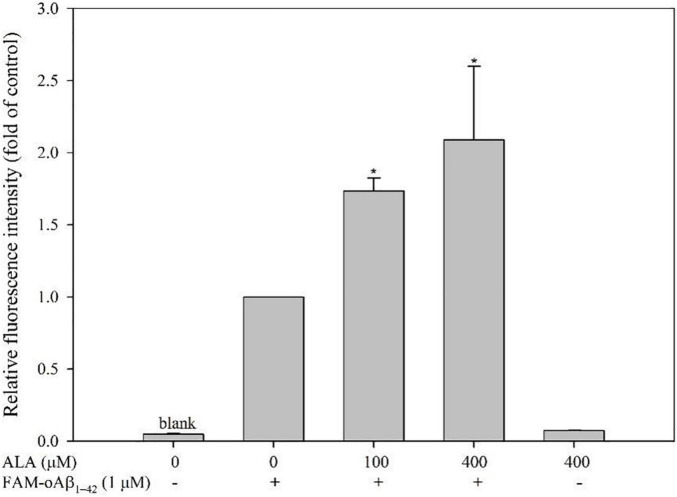
Effect of alpha-lipoic acid (ALA) on 5(6)-carboxyfluorescein (FAM)-oligomeric Aβ (oAβ_1–42_) uptake in BV-2 mouse microglial cells. BV-2 cells were pretreated with or without ALA (100 or 400 μM) for 30 min, followed by FAM-oAβ_1–42_ treatment for 24 h. Mean fluorescence intensity was detected using a flow cytometer. *p < 0.05 vs. the FAM-oAβ_1–42_ group.

The fluorescence of BV-2 cells increased by 73.44 ± 8.99% under 100 μM ALA treatment (*p* < 0.05) and fluorescence intensity increased by 108.83 ± 51.07% under 400 μM ALA treatment (*p* < 0.05) compared with the ALA untreated group, which demonstrated a dose-dependent effect. The 400 μM ALA-treated group with untreated FAM-oAβ_1–42_ showed no significant difference in fluorescence from that of the blank group. Therefore, we confirmed that the increase in fluorescence of the BV-2 cells by ALA was not attributed to ALA itself—rather it was caused by an increased uptake of FAM-oAβ_1–42_ (Figure 2).

### Messenger RNA Expression and oAβ1-42 Phagocytosis-Related Receptors in BV-2 Cells

**FIGURE 3:**
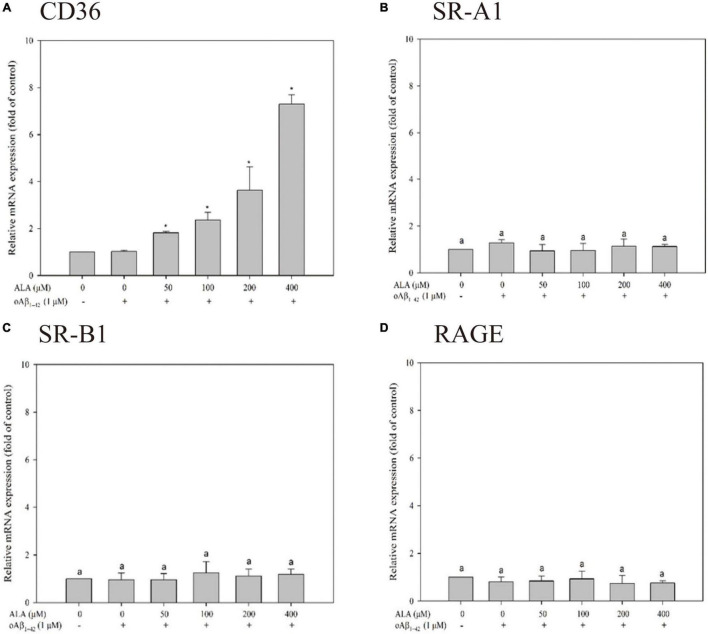
Messenger RNA (mRNA) expression of oAβ_1–42_ phagocytosis-related receptor including CD36 **(A)**, scavenger receptor A1 (SR-A1) **(B)**, scavenger receptor B1 (SR-B1) **(C)**, and receptor for advanced glycation endproducts (RAGE) **(D)** in BV-2 mouse microglial cells. BV-2 cells were pretreated with or without different concentrations of ALA for 30 min, followed by oAβ_1–42_ treatment for 24 h. The relative mRNA expression of CD36, SR-A1, SR-B1, and RAGE was detected using real-time PCR. *p < 0.05 vs. the control group.

**FIGURE 4:**
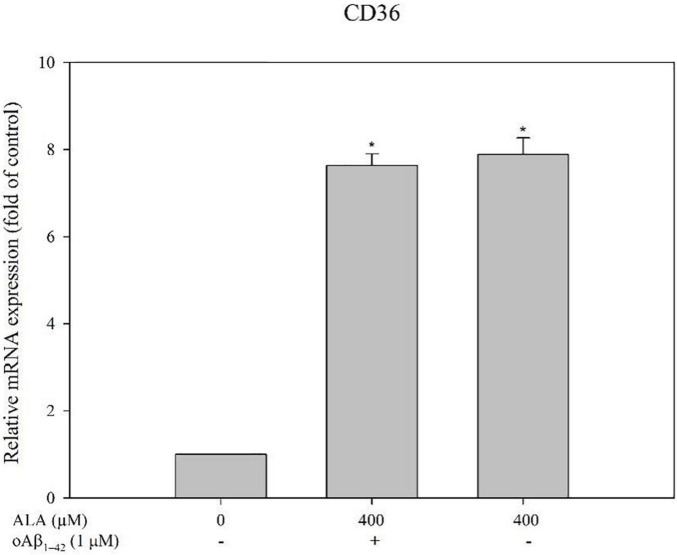
Alpha-lipoic acid elevated CD36 mRNA expression in BV-2 mouse microglial cells. BV-2 cells were pretreated with or without ALA (400 μM) for 30 min, followed by vehicle or oAβ_1–42_ treatment for 24 h. The relative mRNA expression of CD36 was detected using real-time PCR. *p < 0.05 vs. the control group.

Cells were treated with 50, 100, 200, and 400 μM ALA compared with the untreated ALA and oAβ group (control group); mRNA expression levels of CD36 were 1.81 ± 0.06, 2.36 ± 0.33, 3.63 ± 1.00, and 7.29 ± 0.39 times for the respective ALA treatment groups (50, 100, 200, and 400 μM; *p* < 0.05) (Figure 3A). However, mRNA expression levels of SR-A1, SR-B1, and RAGE showed no noticeable dose-dependent effect (Figures 3B–D). In addition, ALA increased the expression of CD36 mRNA in BV-2 cells that were not treated with oAβ_1–42_ (*p* < 0.05); this result was similar to that of the group treated with 1 μM oAβ_1–42_ (*p* < 0.05) (Figure 4).

**FIGURE 5:**
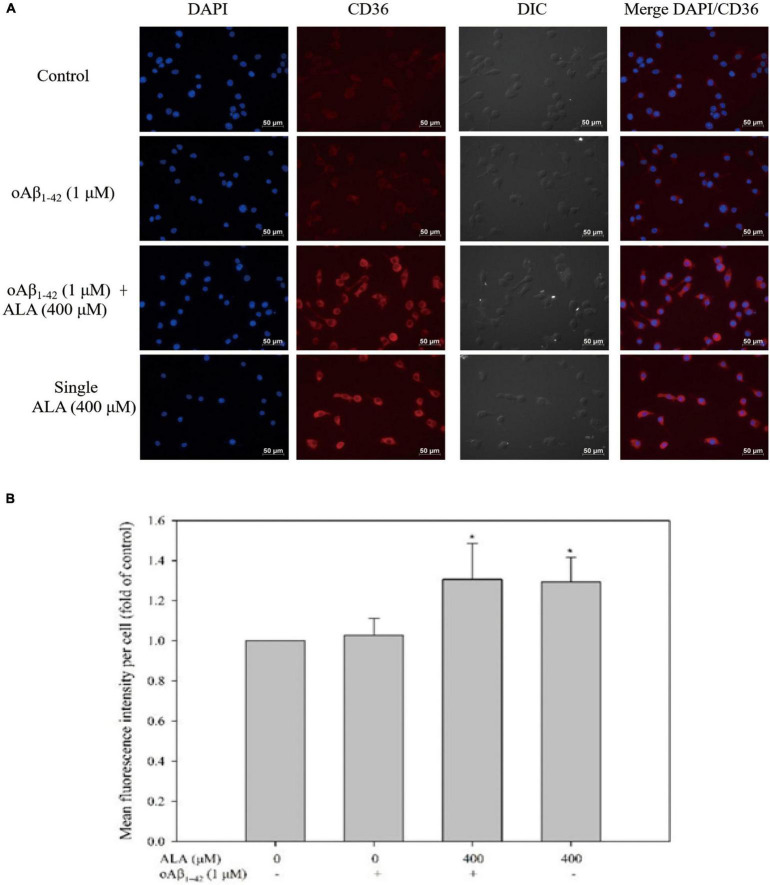
Alpha-lipoic acid elevated CD36 protein expression in BV-2 mouse microglial cells. BV-2 cells were pretreated with or without ALA (400 μM) for 30 min, followed by vehicle or oAβ_1–42_ treatment for 24 h. After treatment, BV-2 cells were immunostained with CD36 antibody (red) and the nuclei were stained with DAPI (blue). **(A)** Fluorescence image from immunocytochemistry. **(B)** Quantification of mean fluorescence intensity per cell. *p < 0.05 vs. the control group.

The expression of ICC-measured CD36 protein is shown in Figure 5A. When Alexa 647 was used to stain CD36 on the cell membrane, it appeared red in the image, whereas the nuclei of the BV-2 cells appeared blue when stained with DAPI. The appearance of cells was analyzed to confirm the size and location of the cells using differential interference contrast light paths. Cells treated with ALA promoted the protein expression of CD36. Regardless of whether oAβ_1–42_ was stimulated, CD36 protein expression in BV-2 cells increased with ALA treatment. The fluorescence intensity of the 400 μM ALA treatment group was approximately 1.3 times that of the untreated group (*p* < 0.05) (Figure 5B).

**FIGURE 6:**
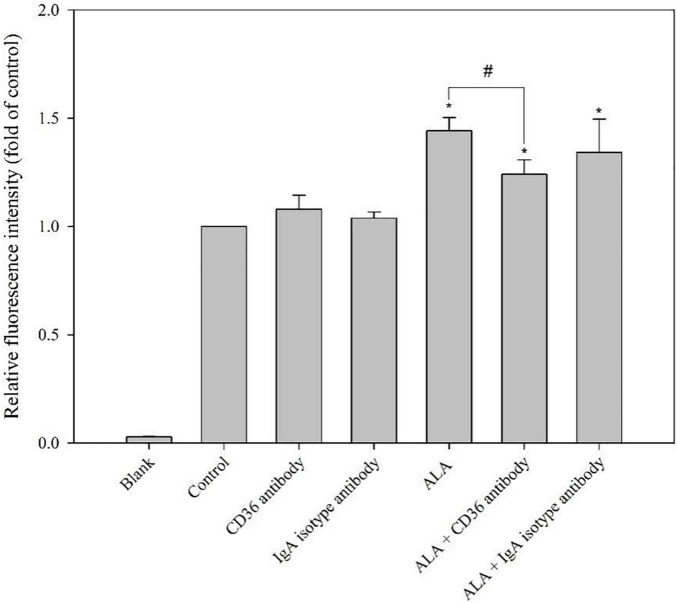
Blocking effect of CD36 antibody on ALA-enhanced phagocytosis ability of FAM-oAβ_1–42_ in BV-2 mouse microglial cells. BV-2 cells were pretreated with or without ALA (400 μM) in the presence of a CD36 antibody or isotype control for 30 min, followed by FAM-oAβ_1–42_ treatment for 24 h. Mean fluorescence intensity was detected using a flow cytometer. *p < 0.05 vs. the control group and ^#^*p* < 0.05 vs. the ALA group.

The addition of the CD36 antibody significantly reduced the uptake capacity of FAM-oAβ_1–42_ enhanced by ALA. The immunoglobulin A isotype control antibody did not affect the uptake of FAM-oAβ_1–42_ by BV-2 cells. In addition, the CD36 antibody did not affect the ability of BV-2 cells without ALA treatment to take up FAM-oAβ_1–42_. Results showed that ALA increased the uptake of oAβ_1–42_ by increasing the expression of CD36 (*p* < 0.05) (Figure 6).

### Alpha-Lipoic Acid Promotes the Translocation of PPAR-γ in BV-2 Cells

**FIGURE 7:**
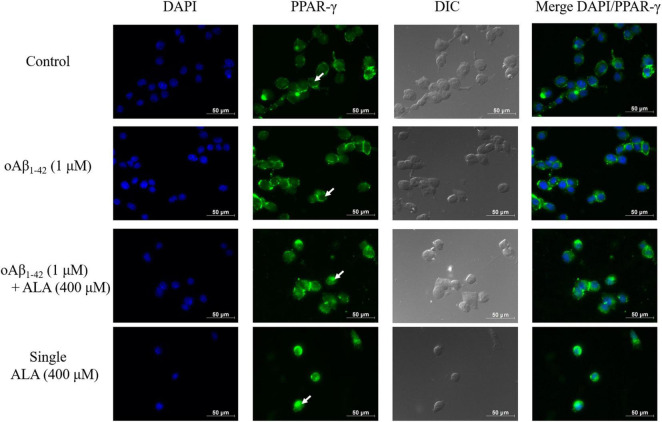
Alpha-lipoic acid caused PPAR-γ translocation from the cytoplasm to the nucleus. BV-2 cells were pretreated with or without ALA (400 μM) for 30 min, followed by vehicle or oAβ_1–42_ treatment for 24 h. After treatment, BV-2 cells were immunostained with PPAR-γ antibody (green) and the nuclei were stained with DAPI (blue).

When Alexa 488 was used to stain PPAR-γ, it appeared green in the image, whereas the nuclei of the BV-2 cells appeared blue when stained with DAPI, as shown in Figure 7. In cells without ALA treatment, most PPAR-γ were located outside the nuclei. Furthermore, cavities were evident in the nucleus, as shown by the white arrow in Figure 7. However, in cells treated with ALA, the transcription factor PPAR-γ was translocated and transferred from the cytoplasm to the nucleus and the void area in the nucleus was significantly reduced compared to that of the untreated group. This was observed regardless of whether oAβ_1–42_ was stimulated.

### Alpha-Lipoic Acid Causes PPAR-γ Translocation Through COX-2/15-Deoxy-^Δ12,14^-PGJ2 in BV-2 Cells

**FIGURE 8:**
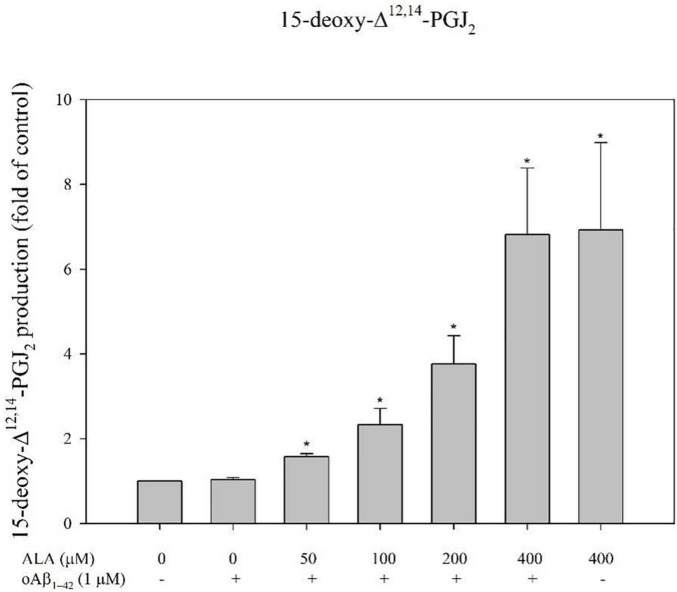
Alpha-lipoic acid promoted 15-deoxy-^Δ12,14^-PGJ2 production in BV-2 mouse microglial cells. BV-2 cells were pretreated with or without different concentrations of ALA for 30 min, followed by vehicle or oAβ_1–42_ treatment for 24 h. Relative 15-deoxy-^Δ12,14^-PGJ2 production was detected using ELISA. Data are presented as means ± SDs. *p < 0.05 vs. the vehicle control group.

Alpha-lipoic acid promoted BV-2 cells and increased the production of 15-deoxy-^Δ12,14^-PGJ2 for the respective ALA treatment groups (50, 100, 200, and 400 μM; *p* < 0.05) (Figure 8). For example, in BV-2 cells that were treated with 400 μM ALA for 24 h, the amount of 15-deoxy-^Δ12,14^-PGJ2 produced was 6.92 ± 2.06 times that of the untreated group and there was no significant difference from the ALA group cocultured with oAβ_1–42_ (Figure 8).

**FIGURE 9:**
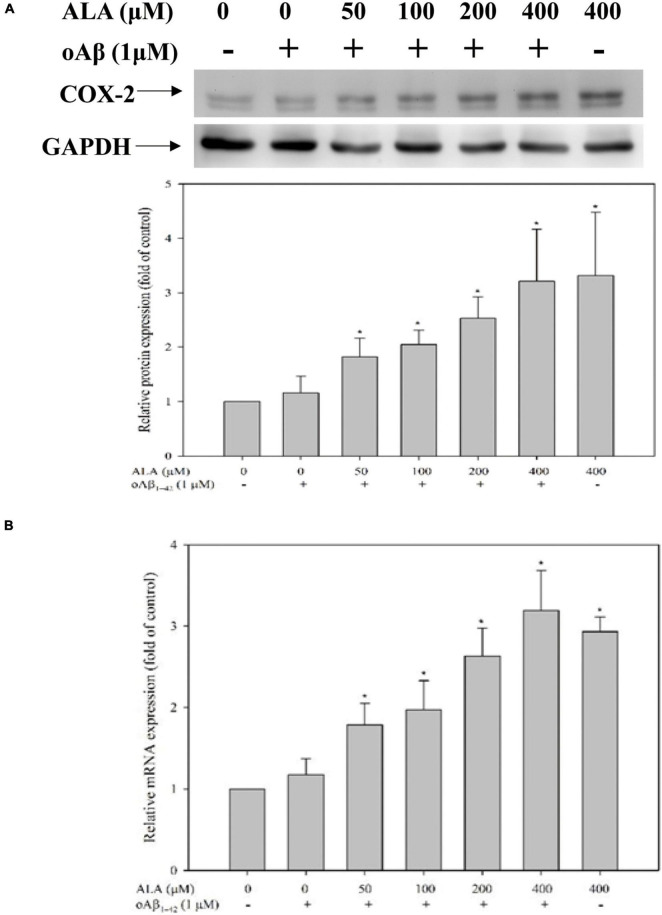
Alpha-lipoic acid elevated cyclooxygenase-2 (COX-2) expression in BV-2 mouse microglial cells. BV-2 cells were pretreated with or without different concentrations of ALA for 30 min, followed by vehicle or oAβ_1–42_ treatment for 24 h. **(A)** The relative COX-2 protein expression was detected using western blotting. **(B)** The relative mRNA expression of COX-2 was determined using real-time PCR. *p < 0.05 vs. the vehicle control group.

After cocultured with oAβ_1–42_ in BV-2 cells for 24 h, the expression of the COX-2 protein did not differ significantly from that of the untreated oAβ_1–42_ group. However, the expression of the COX-2 protein in BV-2 cells increased with the increase in ALA treatment concentration (*p* < 0.05) (Figure 9A). In addition, the expression of COX-2 mRNA in BV-2 cells was similar to the expression of the protein (Figure 9B).

## Discussion

**FIGURE 10:**
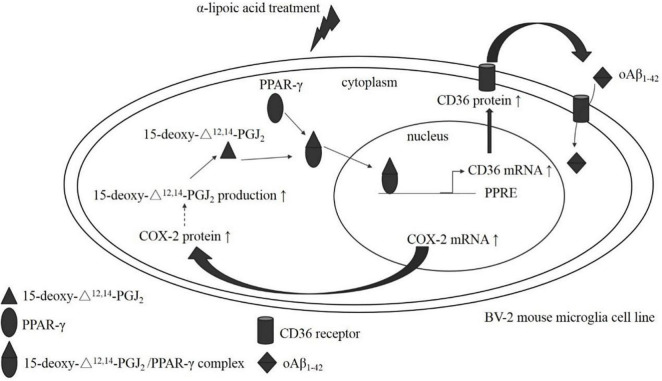
The postulated mechanism of ALA in enhancing oAβ_1–42_ phagocytosis in BV-2 mouse microglial cells. BV-2 mouse microglial cells made a difference after ALA stimulation, which caused the elevation of COX-2 mRNA and the increased translation of the COX-2 protein. Furthermore, the COX-2 protein induced 15-deoxy-^Δ12,14^-PGJ2 production and the 15-deoxy-^Δ12,14^-PGJ2 acted on the PPAR-γ ligand. Simultaneously, the 15-deoxy-^Δ12,14^-PGJ2/PPAR-γ complex translocated from the cytoplasm to the nucleus. PPAR-γ that located the nucleus stimulated the transcription of the CD36 gene, which promoted the synthesis of CD36 mRNA. The increase in CD36 mRNA promoted the translation of the CD36 protein. Finally, the elevated level of the CD36 protein enhanced the phagocytosis of oAβ_1–42_ in BV-2 mouse microglial cells.

This study demonstrated the possible mechanism of ALA on promoting oAβ_1–42_ uptake in mouse microglial BV-2 cells. ALA treatment increased both the expression of COX-2 mRNA and the COX-2 protein in BV-2 cells. The increase in COX-2 protein expression indirectly accelerated the production of 15-deoxy-^Δ12,14^-PGJ2 and acted as a PPAR-γ transcription factor ligand, which resulted in the translocation and combination of PPAR-γ with peroxisome proliferator hormone response elements. In addition, ALA increased the transcription of CD36 mRNA and increased the translation of CD36 mRNA to the CD36 receptor, which recognized oAβ_1–42_ and triggered the phagocytosis of oAβ_1–42_ in BV-2 cells (Figure 10). Thus, our findings suggested that ALA possesses significant potential for regulating the uptake of FAM-oAβ_1–42_ in BV-2 cells. A previous study demonstrated that supplementing 600 mg ALA per day ameliorated the progression of AD (Hager et al., 2007; Fava et al., 2013). However, the mechanism is not well understood. Numerous studies on the alleviating effect of ALA on AD have focused on its antioxidative and anti-inflammatory properties (Maczurek et al., 2008; Farr et al., 2012; Dos Santos et al., 2019; Kim et al., 2019; Choi et al., 2020). We revealed that in addition to antioxidant and anti-inflammatory properties, ALA may also enhance the ability of microglia to eliminate oAβ_1–42_ and subsequently reduce the neurotoxic effect induced by oAβ_1–42_, which may offer a possible mechanism underlying the prevention of dementia.

It is well established that phagocytosis of oAβ by microglia is accomplished *via* a variety of receptors on the cell membrane such as CD36, SR-A1, SR-B1, and RAGE (Yan et al., 1996; El Khoury et al., 1998; Sokolowski and Mandell, 2011). The scavenger receptor CD36 has been identified as essential in the Aβ-recognition microglia activation (Doens et al., 2017; Dobri et al., 2021). In this study, the mRNA expression of CD36 increased with the increase in the concentration of ALA. We speculate that ALA increases phagocytosis of oAβ_1–42_ in BV-2 cells *via* the CD36 receptor. Additionally, the increase in CD36 mRNA expression in BV-2 cells treated with ALA did not appear to be related to oAβ_1–42_. In this study, ALA inhibited CD36 receptor expression and subsequently the uptake of FAM-oAβ_1–42_, but was unable to reverse this progression in BV-2 cells. We speculate that this may be executed solely through CD36, alongside the involvement of other receivers such as Toll-like receptor (TLR)-4 and TLR6 (Stewart et al., 2010). CD36 activation by different ligands is another reason; ligands of microglia in rodents are apoptotic bodies and oxidized low-density protein (Dobri et al., 2021). The CD36 antibody did not inhibit the uptake of FAM-oAβ_1–42_ in BV-2 cells, which is consistent with a previous study (Yang et al., 2011). Increasing the expression of CD36 may promote the ability of microglia to take up Aβ. Results of the Morris water maze test in transplant amyloid precursor protein 23 (APP23) mice showed that the Aβ deposition in the brains of mice in the interleukin-4/interleukin-13 (IL-4/IL-13) treatment group was lower than that in the control group and spatial learning and memory abilities improved significantly more in the treatment group compared with the untreated group, which may be attributed to the increased expression of CD36 by microglia (Kawahara et al., 2012). *In vitro*, it was found that increased expression of CD36 promoted the uptake of oAβ_1–42_ in rat primary type 2 microglia treated with IL-4 (Shimizu et al., 2008). Therefore, we speculate that ALA increases CD36 expression and, subsequently, the CD36 receptor identifies oAβ_1–42_ and initiates downstream phagocytosis.

Peroxisome proliferator-activated receptor-γ has been confirmed as a transcription factor of CD36 mRNA (Shimizu et al., 2008; Yamanaka et al., 2012). In this study, PPAR-γ moved from the cytoplasm to the nucleus under ALA treatment. Thus, the increase in CD36 mRNA facilitated by ALA is related to the increase in the translocation of transcription factor PPAR-γ. The treatment of ALA may increase the activity of PPAR-γ and prevents a variety of diseases (Golbidi et al., 2011; Wang et al., 2013). In a previous study using a PPAR-γ activator, when pioglitazone and DSP-8658 processed rat primary microglia, the expression of CD36 significantly increased and the increase in the CD36 receptor was shown to be the key receptor for pioglitazone and DSP-8658, which promoted the uptake of Aβ_1–42_ by rat primary microglia (Yamanaka et al., 2012). This illustrated the importance of PPAR-γ/CD36 in regulating the uptake of Aβ_1–42_. PPAR-γ must be combined with its ligand to be translocated. The anti-inflammatory prostaglandin 15-deoxy-^Δ12,14^-PGJ2 produced in COX-2 metabolizing polyunsaturated fatty acids is one of the key ligands that activate PPAR-γ (Scher and Pillinger, 2005; Ulivi et al., 2011; Ramer et al., 2013). To date, there has been no evidence demonstrating that ALA promotes the production of 15-deoxy-^Δ12,14^-PGJ2 in cells. Therefore, we speculate that anti-inflammatory response mechanism of ALA is linked to the production of 15-deoxy-^Δ12,14^-PGJ2 and is involved in PPAR-γ translocation in BV-2 cells. We used the anti-inflammatory properties of ALA to explore PPAR-γ activation and found that BV-2 cells increased the production of 15-deoxy-^Δ12,14^-PGJ2 under ALA treatment. This also increased the upstream expression of COX-2, which showed a trend increase with the increase in ALA treatment concentrations. Furthermore, we found that BV-2 cells increased the production of COX-2 mRNA under ALA stimulation, which suggested that ALA increases the translocation of PPAR-γ in BV-2 cells through COX-2/15-deoxy-^Δ12,14^-PGJ2.

Study with respect to the effect of ALA on COX-2 expression in microglia is limited. ALA is considered to be a substance with anti-inflammatory activity and COX-2 has a close relationship with inflammation (Wang et al., 2018). Diethylnitrosamine and choline-methionine-deficient diet-induced liver damage and ALA administration in Fischer rats showed that ALA increases hepatic COX-2 protein expression when the liver is damaged. However, this result was not found in the choline-supplemented diet group (Perra et al., 2008). Moreover, ALA did not increase the protein expression of COX-2 in RAW 264.7 cells (Hwang et al., 2015). We believe that this issue has been inadequately explored; although prostaglandin produced by COX-2-metabolizing polyunsaturated fatty acids is partially related to the inflammatory response, it is also partially related to the anti-inflammatory effect. Few studies have focused on the ability of ALA itself to induce COX-2. The best example is 15-deoxy-^Δ12,14^-PGJ2, which is a well-known anti-inflammatory prostaglandin and currently, studies on the increase of PPAR-γ translocation by ALA are limited. The results of this study clearly showed a correlation among ALA, 15-deoxy-^Δ12,14^-PGJ2, PPAR-γ, and anti-inflammatory effects. Taken together, we speculate that ALA increases the expression of CD36 *via* COX-2/15-deoxy-^Δ12,14^-PGJ2/PPAR-γ activation, which may be one of the major mechanisms of oAβ phagocytosis in BV-2 cells. A future study should be conducted with an *in vivo* study to confirm our *in vitro* findings.

## Conclusion

This study demonstrated that ALA increases phagocytosis of oAβ_1–42_ possibly *via* modulating the COX-2/15-deoxy-^Δ12,14^-PGJ2/PPAR-γ/CD36 pathway in microglia. We postulated that ALA enhances mRNA and protein expression of COX-2, increases the production of 15-deoxy-^Δ12,14^-PGJ2, acts as a PPAR-γ transcription to elevate CD36 mRNA translation into CD36 protein, and subsequently enhances oAβ_1–42_ phagocytosis in BV-2 cells. These study findings suggest that ALA possesses antidementia potential and offers new insight into the mechanisms underlying the prevention of progression of AD.

## Data Availability Statement

The original contributions presented in the study are included in the article/supplementary material, further inquiries can be directed to the corresponding author.

## Author Contributions

C-YK and S-CS participated in the design of the study and writing the protocol. Y-WC carried out all the experiments. C-YK, J-HX, YL, JW, W-CH, and S-CS conducted kinds of literature searches and data analyses and wrote drafts of the manuscript. All authors contributed to the article and approved the submitted version.

## Conflict of Interest

The authors declare that the research was conducted in the absence of any commercial or financial relationships that could be construed as a potential conflict of interest.

## Publisher’s Note

All claims expressed in this article are solely those of the authors and do not necessarily represent those of their affiliated organizations, or those of the publisher, the editors and the reviewers. Any product that may be evaluated in this article, or claim that may be made by its manufacturer, is not guaranteed or endorsed by the publisher.

## Footnotes

1
https://sedfitsedphat.nibib.nih.gov/software/default.aspx
